# A new perspective on studying burial environment before archaeological excavation: analyzing bacterial community distribution by high-throughput sequencing

**DOI:** 10.1038/srep41691

**Published:** 2017-02-07

**Authors:** Jinjin Xu, Yanfei Wei, Hanqing Jia, Lin Xiao, Decai Gong

**Affiliations:** 1 Department of History of Science and Scientific Archaeology, University of Science and Technology of China, Hefei, 230026 China; 2 Jingzhou Preservation Centre of Cultural Relics, Jingzhou, 434020 China; 3 Jingzhou Museum, Jingzhou, 434020 China; 4 Chengdu Institute of Cultural Relics and Archaeology, Chengdu, 610000 China

## Abstract

Burial conditions play a crucial role in archaeological heritage preservation. Especially, the microorganisms were considered as the leading causes which incurred degradation and vanishment of historic materials. In this article, we analyzed bacterial diversity and community structure from M1 of Wangshanqiao using 16 S rRNA gene amplicon sequencing. The results indicated that microbial communities in burial conditions were diverse among four different samples. The samples from the robber hole varied most obviously in community structure both in Alpha and Beta diversity. In addition, the dominant phylum in different samples were *Proteobacteria, Actinobacteria* and *Bacteroidetes*, respectively. Moreover, the study implied that historical materials preservation conditions had connections with bacterial community distribution. At the genus level, *Acinetobacter* might possess high ability in degrading organic culture heritage in burial conditions, while *Bacteroides* were associated closely with favorable preservation conditions. This method contributes to fetch information which would never recover after excavation, and it will help to explore microbial degradation on precious organic culture heritage and further our understanding of archaeological burial environment. The study also indicates that robbery has a serious negative impact on burial remains.

A large amount of archaeological and cultural heritage is preserved in the soil or wet environment around the world. It is well recognized that burial conditions play a crucial role in archaeological heritage preservation, particularly for the organic remains dated back to thousands of years^1^. While research into organic remains in burial environment has been in progress^2^,^3^, the number of such studies is still limited, especially in microbial degradation. Microorganisms were considered as the primary organic decay agents within the soil environment^4^. However, due to a lack of effective methods and typical samples, very few studies were carried out to verify the hypothesis and explore the mechanism.

Microbial community variations in buried soils can be investigated with the development of molecular fingerprinting techniques such as denaturing gradient gel electrophoresis (DGGE)^5^, but the number of bands on the DGGE gel is less, which limits the overall understanding of microbial community composition^6^. However, high-throughput sequencing technologies have opened new frontiers in microbial community analysis and been used widely in evaluating the diversity of soil bacterial components^7^. The recent application of the next-generation sequencing methods such as Illumina and Roche provide a more direct way to detect the microbial taxa^8^,^9^. Studies of 16 S rRNA give insight into which microbial taxa are present in a given sample because it is an excellent phylogenetic marker^10^. So it may be a feasible way to explore archaeological burial conditions from the perspective of microorganisms.

Jingzhou, where used to be the capital of a formidable country Chu Kingdom during the Warring States (475–221 BC) in China, was a miraculous place for ancient silk and lacquer preservation^11^. Gorgeous silk fabrics were unearthed in two-thousand-year tombs such as M1 of Mashan and M2 of Baohe^12^,^13^. Located in southern region of China, Jingzhou’s humidity climate makes it hard to preserve organic materials underground. However, the survival of the organic remains raised the concerns on studying the burial environment.

The microbial communities may change immediately upon the excavation. Later, the precious environment information will disappear irreversibly. So the representative samples collected from the undisturbed burial environment are the key to ensure the reliability of the results. Our samples were excavated from No. 1 Wangshanqiao Chu tomb (475–221BC) in Jingzhou, Hubei Province in 2015, using standard coring methods.

The objective of this work is to analyze bacterial diversity and community structure from M1 of Wangshanqiao using 16 S rRNA gene amplicon sequencing. To our knowledge, this method hasn’t been reported in the study of archaeological burial environment. It is expected to distinguish diversity of bacterial communities *in situ* to find potential important cultural information and develop a better understanding of archaeological remains, as well as explore the influence of microorganisms on degradation.

## Results

### Soil and water physicochemical properties

The soil physicochemical properties were presented in Table 1. WS4 was water sample so some physicochemical properties weren’t detected. Soil moisture changed notably from 28.57 to 45.32 because of precipitation and surface water influence on WS3. Soil pH value ranged from 6.98 and 7.37. It can be explained that the raw soil was close to neutralization but the precipitation was slightly alkaline. Furthermore, ancient people may artificially add lime to the caesious mud called Qinggaoni, which can be inferred by the high concentrations of calcium in WS3 and WS4. Soil salinity was lower in all samples compared with agricultural soil. WS2 revealed lowest carbon and nitrogen contents while WS3 displayed highest carbon contents. However, the results suggested that most of physicochemical properties varied slightly, while some abnormal data can be explained by special microenvironment created by man-made.

### Microbial diversity and community structure

After quality filtering the raw reads, a total of 613299 effective sequences were recovered from all 12 samples (replicates contained). Each library contained 22567 to 112681 reads, while different phylogenetic OTUs (determined at ≥97% similarity) were in the range of 230–447, with WS3–3 having the highest and WS2–1 having the lowest.

The alpha diversity indices for all samples were shown in Table 2, of which the Chao1 and ACE estimators reveal the community richness and Shannon and Simpson index indicate the community diversity. The results showed that WS2 achieved significantly lowest index of Chao1, ACE, Shannon and Simpson, suggesting that WS2 was different from other samples in microbial richness and diversity. Both of them were lower in WS2, while the other three groups shared approximate microbial richness and evenness. Rarefaction curves of Chao1 tended to approach the saturation plateau, which indicated the results reasonable.

**Figure 1:**
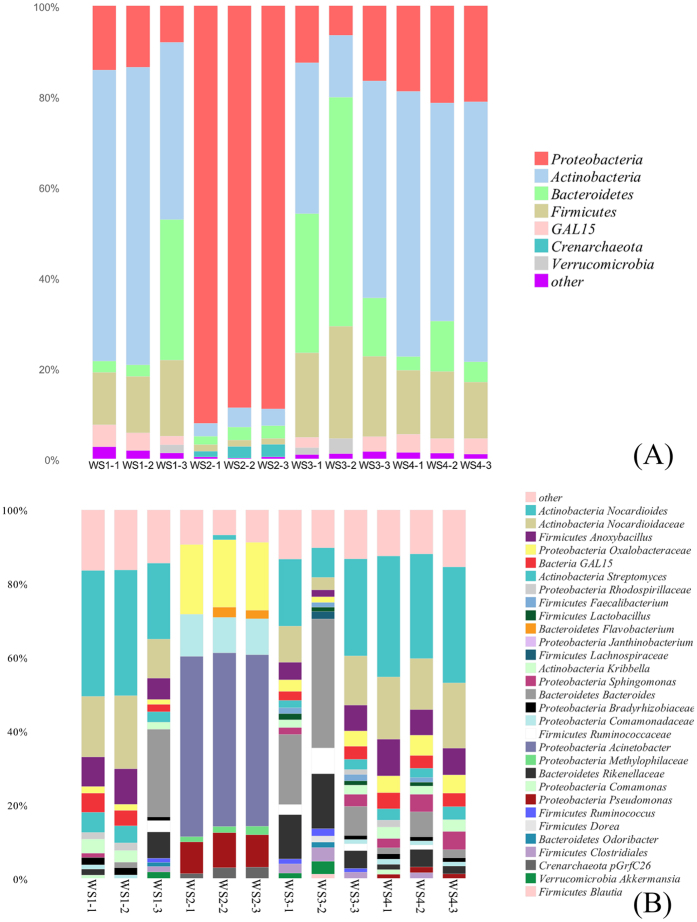
Microbial community at phylum (**A**) and genus (**B**) level. The OTUs whose proportion was less than 1% was assigned as other. Refer to Table 3 for sample abbreviations.

The sequence of different samples were classified at different taxonomic levels using RDP classifier. There was no significant difference in kingdom level, where Bacteria occupied at least 97% population. However, the histogram (Fig. 1) witnessed marked shift at the phylum and genus level, especially in sample group WS2. The composition of microbial communities included 6 different bacterial phyla (*Proteobacteria, Actinobacteria, Bacteroidetes, Firmicutes, Verrucomicrobia, GAL15)* with selecting the relative abundance above 1%. Microbial community exhibited little difference in diversities but large in abundances at Phylum level. *Proteobacteria* was the most abundant group in WS2, accounting for approximate 90% of total bacteria. While *Actinobacteria* was the dominant phyla in WS1 and WS4, followed by *Proteobacteria.* Larger percentages of *Bacteroidetes* (31.41%) and *Actinobacteria* (31.62%) were observed in WS3. When it comes to genus level, *Acinetobacter* comprised nearly 50% of the OTUs in WS2, but was found less than 0.1% relative abundance in microbial distribution of other samples. *Bacteroides* played a predominant role in microbial structure of WS3. Meanwhile, samples of WS1 and WS4 still had the most frequently genus *Nocardioides* in common, representing about 30% of the OTUs. Generally speaking, taxonomic distribution varied strikingly in WS2 compared with other samples, and indicated higher similarity in WS1 and WS4.

**Figure 2:**
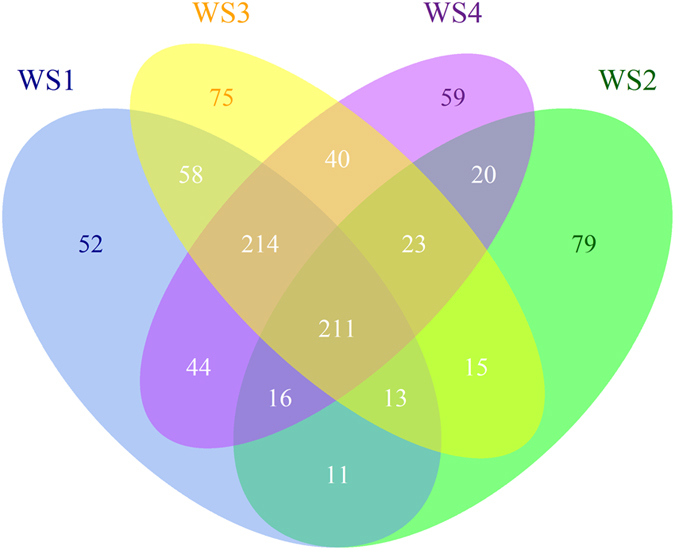
Venn Drawing showing the unique and shared OTUs of all samples. The three replicates were calculated and exhibited as a single sample named WS1, WS2, WS3, and WS4.

At the genus level, observed differences between samples were represented in Fig. 2. The number of shared OTUs among these samples was 211. WS2 and WS3 possessed more unique OTUs than WS1 and WS4. The largest overlap was detected between WS1 and WS3, followed by WS3 and WS4, while the smallest overlap was found between WS1 and WS2.

**Figure 3:**
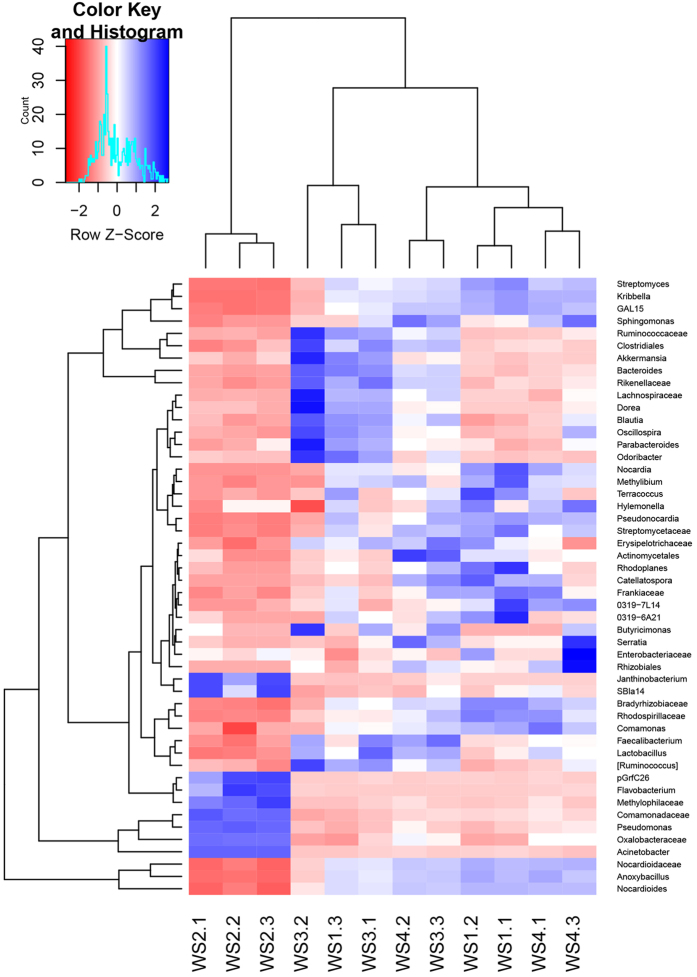
Microbial distribution of the top 50 abundant genera. The high abundant was shown with blue and the low abundant was shown with red.

The top 50 abundant genera in all samples were selected as the representative genera shown in the heat map (Fig. 3). There was a significant variation of microbial community between WS2 and other samples. The other samples had close connection with each other so that some of repetitions were separated. The dominant genera in WS3-1, WS3-2 and WS1-3 were different from the rest, while W1-1 and W1-2 gathered firstly with WS4-1 and WS4-3.

**Figure 4:**
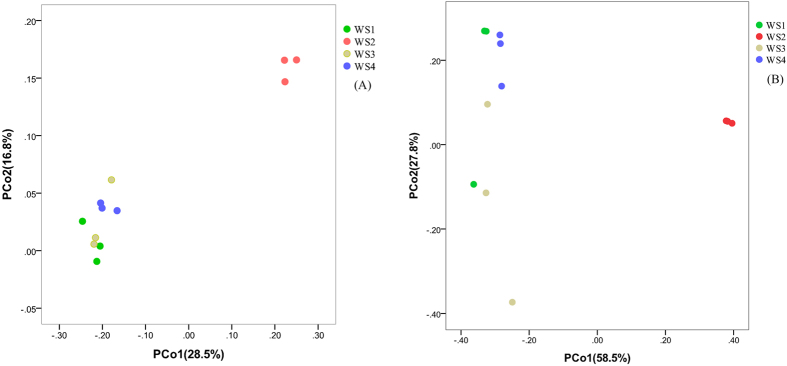
Bacterial communities by principal coordinate analysis (PCoA). The chart (**A**) was based on unweighted unifrac distance matrix and the chart (**B**) was based on weighted unifrac distance matrix.

Principal Co-ordinates Analysis which was shown in Fig. 4 is a universally used method in analyzing Beta diversity of microbial distribution. PCoA based on unweighted unifrac distance matrix and weighted unifrac distance matrix revealed clear distinctions between bacterial populations between different samples, respectively. Samples WS2 grouped together, whereas the other samples had no clear boundary.

Although the microbial communities were diverse obviously, physicochemical properties of different samples differed slightly. However, the surprising difference in WS2 had close relationship with the robbery situation, which may affect the results strongly.

## Discussion

Bacteria inhabiting in the soil subsurface plays a vital role in organic matter decomposition, especially when soil depth is increasing^14^. In this work, soil and liquid bacteria community structure of the No. 1 Wangshanqiao Chu tomb were studied using 16 S rRNA gene amplicon sequencing, which broadens the understanding of burial conditions.

Contamination was effectively avoided in the samples by using standard coring methods, which guaranteed the reliability. The depths of samples included 4 m and 10 m, 11 m, which overstepped the general study range in many related research field like agriculture and environmental sciences^15^,^16^. Many factors including low temperature, less oxygen and nutrient availability restricted microbial activity in the deeper soil horizons. Previous studies have thought fewer microorganism can survive under these circumstances^17^. However, the overall microbial communities in burial conditions were unexpectedly diverse, harboring a wide variety of taxa, some of which was common in soil environment^18^. Since the bacterial taxa data is short in archaeological burial conditions, comparative study of community structures can be difficult. It is noteworthy that this burial environment is similar to that of sediment and permafrost. A large number of research were reported on these bacteria with unique function^19^,^20^, which provides information for our study.

The results indicated that bacterial communities in different samples were surprisingly diverse, which had significant implication of perceiving biodegradation underground. The samples from the robber hole (WS2) varied obviously in community structure both in Alpha and Beta diversity. The predominant phylum in them was *Proteobacteria*, which differed obviously from others. *Proteobacteria* which was observed frequently to take the lead in soils^18^,^21^, is of great importance to global carbon and nitrogen cycling^22^. As nutritional bacteria, it can utilize complex organic matter to metabolize. Moreover, the genus *Acinetobacter* accounted for nearly the half in the samples from the rob hole. This bacteria is ubiquitous microorganism in soil, water, and sewage^23^, and was referred to occupy nearly 0.001% of the total heterotrophic aerobic population of soil and water^24^. Much research has reported efficient degradation ability of *Acinetobacter*, widespreadly found not only in decomposing cellulose, hemicelluloses, lignin, but also in cleansing waste water and petroleum pollution^25^,^26^,^27^. For instance, an *Acinetobacter* sp. strain was isolated from the fluid steeping with the erosive bamboo slips that were unearthed from the archaeological site of Three Kingdoms in China^28^. *Comamonadaceae* and *Pseudomonas* which occupied in the third and fourth place in the whole population were also reported in studies on the microbial degradation of lignin^28^,^29^. Especially, *Pseudomonas* was implicated in the process of influencing the crystalline structure of cellulose microfibrils so that it was closely associated with the decay of wooden relics^30^. As a result, it can be inferred that the predominant bacteria detected in WS2 would play a vital role in degrading organic culture heritage in burial conditions.

Undisturbed caesious mud showed an abundant proportion of *Bacteroidetes*, which is widely distributed in the environment, within soil, sediments, and sea water. Previous studies suggested that the phylum *Bacteroidetes* makes up an average of 5% of soil bacterial communities^18^. What is remarkable is that *Bacteroidetes* is one of the phylum that was frequently associated with permafrost soils^31^. Moreover, *Bacteroides* which played a predominantly role at the genus level is an important anaerobic member of the gut microbiome^32^ and seemed to be rare in soils. *Firmicutes* was another phylum abundant in undisturbed caesious mud. However, it is deduced that these spore-forming species might be well adapted to cold conditions since it was frequently discovered in permafrost-like habitats^33^,^34^. Furthermore, it must be stressed that *Actinobacteria* was the most abundant group in raw soil and liquid as well as in undisturbed caesious mud. *Actinobacteria* was previously found with a high abundance in permafrost^31^, which was thought to be caused by their maintenance of metabolic activity and DNA repair mechanisms at low temperatures^35^. *Nocardioides* took the lead in raw soil and liquid at the genus level. It is noteworthy that *Streptomyces* accounted for nearly 5% in raw soil while the proportion was few in caesious mud and liquid. *Streptomyces* was found to have efficient degradation ability of lignin by *in vitro* assays^36^.

There are several excavated historical tombs famous for their luxuriant organic archaeological remains such as fragile silk, lacquer in humid region of China. These tombs concentrated regularly on specific area like Jingzhou and Changsha, which attracted archaeologists to search for answer. Generally speaking, silk and other organic matter excavated from No. 1 Wangshanqiao Chu tomb weren’t preserved well. But the preservation conditions varied with shifts on microenvironment. The straw mat near undisturbed caesious mud was better preserved. Silk fabrics found in caesious sludge soil degraded seriously, losing their mechanical strength and even turning into powder, while silk fabrics floating in outer coffin liquid were much better.

The study indicated that burial cultural heritage preservation conditions has connections with their bacterial community distribution. We can speculate that burial conditions of No. 1 Wangshanqiao Chu tomb was favourable for organic matter maintenance at the beginning with anaerobic environment according to the experimental results and previous archaeological discovery nearby. The bacterial tended to degrade slowly in low-oxygen environments and the dominant species may have less capability in degrading organic material like wood and silk. Nevertheless, when the balance was broken due to robbery, the community structure changed observably. *Proteobacteria* which possessed high degradation ability dominated in whole bacterial, with the degree of biodeterioration aggravating. Later, precipitation seeped into the tomb and was filled with the whole outer coffin. The water may be filtered by soil and then cleansing slowly during a long time, leaving microbial community observed nowadays similar to the raw soil and protecting some precious organic historical relics.

Our current knowledge on molecular microbial diversity in burial conditions is extremely poor. The soil microbial community has arguably the highest level of prokaryotic diversity of any environment^37^. Currently, less than 1% of this diversity can be cultivable by traditional techniques^38^. Fortunately, high-throughput sequencing technologies provide a new method to study the burial environment. We attempted to distinguish the bacteria community distribution in a case study applying 16 S rRNA gene amplicon sequencing and drew interesting conclusions successfully.

This method contributes to fetch information which would never recover after excavated. Firstly, the information will play an important role in research on degradation mechanism of precious culture heritage both organic matter and metals in the future, and thus explore potential cultural information of which the value may be underestimated nowadays. The ignored information would contribute to explain complex archaeological phenomenon. For instance, the results indicated that ancient people had realized how to utilize burial conditions to prevent decomposition. Secondly, this method evaluates likelihood of survival about organic matter like silk fabrics in terms of microbial activity. Some indicator may predict where burial microbial condition is likely to preserve treasures before excavation. Thus, it will lead to select appropriate conservation in archaeological site according to predicting objects condition before excavation, which will benefit conservation and cost reduction. Thirdly, the study indicates that robbery has a serious negative impact on burial remains, far more than taking away treasures. Further study can draw public attention towards preservation. Moreover, the results can assess if rob hole reaches the aim successfully before excavation. Lastly, the large organic artifacts tend to reburial *in situ* for protection nowadays in lack of economic resources for excavation and conservation, especially in Europe^39^,^40^. It is no doubt that data of burial microbial environment will provide reference for management.

## Methods

### Sample Site

**Figure 5:**
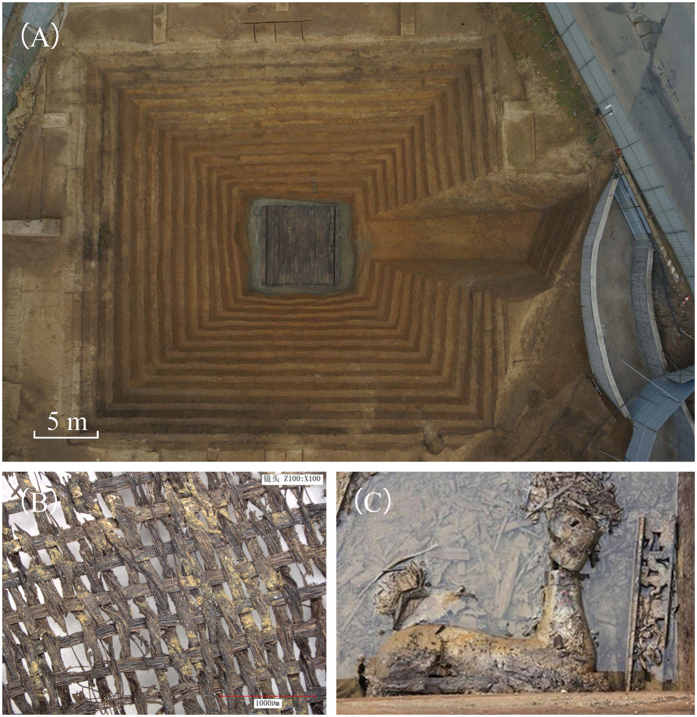
Photographs of No. 1 Wangshanqiao Chu tomb in China. (**A**) Photographs of No. 1 Wangshanqiao Chu tomb. (**B**) Silk fabrics unearthed from No. 1 Wangshanqiao Chu tomb. (**C**) Lacquer buried in outer coffin from No. 1 Wangshanqiao Chu tomb.

The study took place at the No. 1 Wangshanqiao Chu tomb in Jingzhou, Hubei Province. The tomb was built about 2300 years ago in Warring States. Jingzhou witnessed a Golden Age of Chu Kingdom. Plenty of sites of Warring States were found in Jingzhou, and many large noble tombs were also buried here. No. 1 Wangshanqiao Chu tomb is one of the highest grade and largest scale Chu noble tombs that has ever been excavated. It locates in the lower Yangtze region. Figure 5 shows the appearance of the tomb and the arrangement is a typical representation of Chu tomb. However, the large tomb was seriously robbed around Qin-Han Dynasties which caused the turbid water to flow into and stuff the chamber, but a large amount of exquisite cultural relics still survived including bronze, lacquer, jade, textile^41^. Renowned historical tombs were discovered in the district nearby, including No. 1 Mashan Chu tomb which is called treasure-house filled with silk and No. 1 Wangshan Chu tomb which is well-known for Sword of Goujian. Unfortunately, some organic matter, for instance, the silk that was unearthed from No. 1 Wangshanqiao Chu tomb is influenced by robbery (Fig. 5). Although burial environment were disturbed in one spot, the previous burial conditions which are considered as favourable soil conditions for the preservation of archaeological remains and far away from robber hole have been well-retained before excavation. Hence this tomb is a superexcellent case for our study.

**Figure 6:**
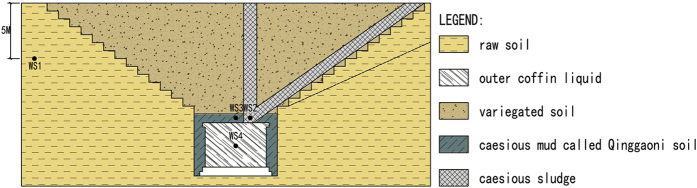


Undisturbed soil was sampled by standard coring methods while scene excavation was moving to the 5 meters above outer coffin. Outer Coffin liquid was sampled once the outer coffin board was slung (Table 3). Detailed plots were marked in Fig. 6. The number of samples is influenced by archaeological excavation limit. Soil and liquid for sequencing analysis were stored at −20 °C, and soil and liquid for other physicochemical properties applications were stored at 4 °C.

### Soil Chemical Analysis

Soil moisture was determined on fresh soil material after drying for 24 h at 105 °C. Soil pH was estimated on 1:2 soil/deionized water extracts which was prepared by passing through a 100 mesh screen. Conductivity of 1:5 soil/water extracts was measured with conductivity meter to determine the soil salinity. Total carbon and total nitrogen contents were measured using an element analyzer (Elementar, Germany).

### DNA Extraction, PCR and 16 S rRNA gene amplicon Sequencing

Total genomic DNA from samples was extracted using FastDNA^®^ Spin Kit for Soil. DNA concentration and quality were checked using a NanoDrop Spectrophotometer. DNA was diluted to 10ng/μl using sterile ultrapure water and stored at −80 °C for downstream use. The 515 F (GTGCCAGCMGCCGCGGTAA) and 806 R (GGACTACHVGGGTWTCTAAT) primers were used to amplify the bacterial 16 S rRNA V4 fragments^7^. The PCR mixture (25 μL) contained 1x PCR buffer, 1.5 mM MgCl_2_, each deoxynucleoside triphosphate at 0.4 μM, each primer at 1.0 μM, 0.5 U of Ex Taq (TaKaRa, Dalian, China) and 10 ng template DNA. The PCR amplification program consists of initial denaturation at 94 °C for 1 min, followed by 30 cycles (denaturation at 94 °C for 20 s, annealing at 54 °C for 30 s, and elongation at 72 °C for 30 s), and a final extension at 72 °C for 5 min. Three replicates of PCR reactions for each sample were combined together. PCR products mixed with 1/6 volume of 6X loading buffer were loaded on 2% agarose gel for detection. Samples with bright main strip between 200–400 bp were chosen for further experiments. The electrophoresis band was purified using OMEGA Gel Extraction Kit (Omega Bio-Tek, USA). DNA was quantified using Qubit@ 2.0 Fluorometer (Thermo Scientific). PCR products from different samples were pooled with equal molar amount. Library preparation and sequencing libraries were generated using TruSeq DNA PCR-Free Sample Prep Kit following manufacturer’s recommendations and index codes were added. The library was then applied to paired-end sequencing (2 × 250 bp) with the Illumina Miseq apparatus at Rhonin Biosciences Co., Ltd.

### Data Analysis

Paired-end reads from the original DNA fragments were merged using FLASH^42^. Paired-end reads were assigned to each sample according to the unique barcode. The sequences with high quality (length >200 bp, without ambiguous base ‘N’, and average base quality score >30) were screened for chimeraschecking using UCHIME algorithm^43^. Sequences were clustered into operational taxonomic units (OTUs) at 97% identity threshold using UPARSE-OUT ref algorithms^44^. Representative sequences were picked for each OTU. Taxonomy were assigned using the greengenes database^45^ and Ribosomal Database Project classifier^46^. Representative sequences were aligned using PyNAST^47^. In case of the influences of sequence depth on community diversity, the OTU table was rarified to make all samples holding the same sequence number. All data analyses were performed using R 3.3.0^48^. Beta diversity metrics were calculated in Vegan^49^. Unweighted and weighted Unifrac distances were calculated in QIIME^50^. Principal Coordinate Analysis (PCoA) were performed using package Ape^51^. Some pictures were made using SPSS19.0 (IBM Corp, Armonk, NY, USA).

## Additional Information

**How to cite this article**: Xu, J. *et al*. A new perspective on studying burial environment before archaeological excavation: analyzing bacterial community distribution by high-throughput sequencing. *Sci. Rep.* **7**, 41691; doi: 10.1038/srep41691 (2017).

**Publisher's note:** Springer Nature remains neutral with regard to jurisdictional claims in published maps and institutional affiliations.

## Figures and Tables

**Table 1:** Physicochemical properties of the soil and water samples.

Properties	WS1	WS2	WS3	WS4
Soil moisture (%)	28.57 ± 0.79	36.45 ± 1.45	45.32 ± 1.33	/
pH	6.98 ± 0.12	7.27 ± 0.18	7.37 ± 0.10	7.31 ± 0.15
Soil salinity (g/kg)	0.15 ± 0.02	0.40 ± 0.02	0.34 ± 0.05	/
C (%)	0.17 ± 0.01	0.34 ± 0.00	0.49 ± 0.01	/
N (%)	0.07 ± 0.01	0.11 ± 0.01	0.11 ± 0.02	/
Ca (μg/ml)	2.09 ± 0.31	3.96 ± 0.11	7.34 ± 0.15	88.58 ± 1.67

**Table 2:** Community richness and diversity estimates for samples.

Sample	OTUs	Chao1	ACE	Shannon	Simpson
WS1-1	399	542	546	4.21	0.934
WS1-2	389	559	568	4.11	0.931
WS1-3	419	574	611	4.32	0.956
WS2-1	230	338	330	3.31	0.885
WS2-2	267	521	512	3.40	0.897
WS2-3	250	366	390	3.42	0.896
WS3-1	443	630	610	4.53	0.965
WS3-2	392	533	546	4.25	0.954
WS3-3	447	575	592	4.55	0.959
WS4-1	409	573	590	4.27	0.940
WS4-2	441	590	622	4.47	0.956
WS4-3	380	490	530	4.30	0.948

**Table 3:** Locations and description of samples.

Sample ID	Sampling location	Depth (m)	Description	Replicate number
WS1	Nearby the tomb	5	WS1 was raw soil.	3
WS2	Robber hole near outer coffin	10	WS2 was caesious sludge soil formed by robbery.	3
WS3	Central point of whole coffin chamber near outer coffin	10	WS3 was undisturbed caesious mud called Qinggaoni usually found in Chu tomb.	3
WS4	Into the outer coffin	11	WS4 was outer coffin liquid.	3
